# Supplementary material for: A cross-sectional pilot study of birth mode and vaginal microbiota in reproductive-age women
Source: PLoS One. 2020 Apr 1;15(4):e0228574. doi: 10.1371/journal.pone.0228574 (PMC7112195; doi:10.1371/journal.pone.0228574)
Supplement: S1 Table — (DOCX) [file pone.0228574.s001.docx]

| **S1 Table: Characteristics of study participants by parent study (N=144)** | | | | | | |
| --- | --- | --- | --- | --- | --- | --- |
|  | HCL  (n=88) | |  | VM400  (n=56) | |  |
|  | n | % |  | n | % | P-value^e^ |
| **Birth mode** |  |  |  |  |  | 0.83 |
| C-section | 16 | 18.2 |  | 11 | 19.6 |  |
| Non-black and non-Latina | 72 | 81.8 |  | 45 | 80.4 |  |
| **Race/ethnicity** |  |  |  |  |  | 0.57 |
| Black or Latina | 30 | 34.1 |  | 19 | 33.9 |  |
| Non-black and non-Latina | 58 | 65.9 |  | 37 | 66.1 |  |
| **Age at study entry** |  |  |  |  |  | 0.001 |
| 17 to 23 | 34 | 38.6 |  | 9 | 16.1 |  |
| 24 to 30 | 38 | 43.2 |  | 23 | 41.1 |  |
| 31 & over | 16 | 18.2 |  | 24 | 42.9 |  |
| **Body mass index ^a^** |  |  |  |  |  | 0.001 |
| ≤24.9 | 43 | 49.4 |  | 31 | 55.4 |  |
| 25.0-29.9 | 14 | 16.1 |  | 19 | 33.9 |  |
| ≥30.0 | 30 | 34.5 |  | 6 | 10.7 |  |
| **Community state type** |  |  |  |  |  | 0.32 |
| CST-I, *L. crispatus*-dominated | 40 | 45.5 |  | 16 | 28.6 |  |
| CST-II, *L. gasseri*-dominated | 1 | 1.1 |  | 2 | 3.6 |  |
| CST-III, *L. iners*-dominated | 25 | 28.4 |  | 21 | 37.5 |  |
| CST-IV, Low *Lactobacillus* | 17 | 19.3 |  | 13 | 23.2 |  |
| CST-V, *L. jensenii*-dominated | 5 | 5.7 |  | 4 | 7.1 |  |
| **Bacterial vaginosis diagnosis within 2 months** |  |  |  |  |  | 0.17 |
| Yes | 6 | 6.8 |  | 1 | 1.8 |  |
| No | 82 | 93.2 |  | 55 | 98.2 |  |
| **Vaginal pH ^b^** |  |  |  |  |  | 0.93 |
| 4.0-4.5 | 61 | 71.8 |  | 40 | 40.4 |  |
| 4.6-5.0 | 12 | 14.1 |  | 9 | 16.1 |  |
| >5.0 | 12 | 14.1 |  | 7 | 12.5 |  |
| **Vaginal symptoms in prior 2 months** |  |  |  |  |  |  |
| Discharge | 3 | 3.4 |  | 16 | 28.6 | <0.001 |
| Itching | 1 | 1.1 |  | 5 | 8.9 | 0.03 |
| **Ever been pregnant** |  |  |  |  |  | 0.14 |
| No | 55 | 62.5 |  | 28 | 50.0 |  |
| Yes | 33 | 37.5 |  | 28 | 50.0 |  |
| **Parity** |  |  |  |  |  | 0.17 |
| 0 | 64 | 72.7 |  | 33 | 58.9 |  |
| 1 | 12 | 13.6 |  | 9 | 16.1 |  |
| 2+ | 12 | 13.6 |  | 14 | 25.0 |  |
| **Ever given birth vaginally** |  |  |  |  |  | 0.07 |
| No | 70 | 79.6 |  | 37 | 66.1 |  |
| Yes | 18 | 20.5 |  | 19 | 33.9 |  |
| **Years since last pregnancy ^b^** |  |  |  |  |  | 0.22 |
| Never pregnant | 55 | 63.2 |  | 28 | 53.6 |  |
| 3 or less | 17 | 19.5 |  | 10 | 18.5 |  |
| more than 3 | 15 | 17.2 |  | 16 | 29.6 |  |
| **Age at menarche ^d^** |  |  |  |  |  | 0.004 |
| ≤12 | 53 | 60.2 |  | 24 | 42.9 |  |
| >12 | 35 | 39.7 |  | 35 | 39.8 |  |
| **Weight status at menarche ^c^** |  |  |  |  |  | 0.65 |
| Average or below | 71 | 80.7 |  | 45 | 80.3 |  |
| Overweight or above | 16 | 18.2 |  | 8 | 14.3 |  |
| **Hormonal contraceptive use (current)** |  |  |  |  |  | 0.73 |
| No | 45 | 51.1 |  | 31 | 55.4 |  |
| Yes | 43 | 48.9 |  | 25 | 44.6 |  |
| **Number of sexual partners in the prior 2 months ^c^** |  |  |  |  |  | 0.32 |
| None | 13 | 15.1 |  | 13 | 24.1 |  |
| 1 | 70 | 81.4 |  | 38 | 70.4 |  |
| 2+ | 3 | 3.5 |  | 3 | 5.6 |  |
| **Douched (ever)** |  |  |  |  |  | 0.03 |
| No | 65 | 73.9 |  | 50 | 89.3 |  |
| Yes | 23 | 26.1 |  | 6 | 10.7 |  |
| **Hygiene product use (2 months)** |  |  |  |  |  |  |
| Feminine towellette | 1 | 3.7 |  | 11 | 9.4 | 0.47 |
| Hygiene spray | 0 | 0.0 |  | 3 | 7.0 | 0.99 |
| Hygiene powder | 1 | 8.3 |  | 2 | 4.8 | 0.54 |
| Other product | 2 | 7.4 |  | 10 | 8.6 | 0.60 |
| **Sanitary product use at last menstrual period ^a^** |  |  |  |  |  | 0.02 |
| Tampon only | 35 | 39.8 |  | 10 | 18.2 |  |
| Sanitary napkin only | 18 | 20.5 |  | 18 | 32.7 |  |
| Tampon and sanitary napkin | 35 | 39.8 |  | 27 | 49.1 |  |
| \| a. Missing for 1 participant; b. Missing for 3 participants; c. Missing for 4 participants;  d. Missing for 5 participants; e. P-values obtained from chi-square or Fisher's exact tests \| \| --- \| | | | | | | |
